# A Unique Response Behavior in the Dissolved Oxygen Tension in *E. coli* Minibioreactor Cultivations with Intermittent Feeding

**DOI:** 10.3390/bioengineering10060681

**Published:** 2023-06-02

**Authors:** M. Adnan Jouned, Julian Kager, Vignesh Rajamanickam, Christoph Herwig, Tilman Barz

**Affiliations:** 1 ICEBE, TU Wien, Gumpendorfer Straße 1a 166/4, 1060 Vienna, Austria; adnan.jouned@tuwien.ac.at; 2 Department of Chemical and Biochemical Engineering, Technical University of Denmark, Building 228A, 2800 Kgs. Lyngby, Denmark; jukager@kt.dtu.dk; 3 Boehringer Ingelheim RCV GmbH & Co KG, Biopharmaceuticals Austria, Dr. Boehringer Gasse 5-11, 1120 Vienna, Austria; vignesh.rajamanickam@boehringer-ingelheim.com; 4 Lisalis GmbH, Jenbachgasse 73-2, 1130 Vienna, Austria; christoph.herwig@lisalis.at; 5 Center for Energy, AIT Austrian Institute of Technology GmbH, Giefinggasse 2, 1210 Vienna, Austria

**Keywords:** dissolved oxygen tension, *E. coli* cultivation, mechanistic model, data-driven analysis, signal analysis

## Abstract

Intermittent bolus feeding for *E. coli* cultivations in minibioreactor systems (MBRs) profoundly affects the cell metabolism. Bolus feeding leads to temporal substrate surplus and transient oxygen limitation, which triggers the formation of inhibitory byproducts. Due to the high oxygen demand right after the injection of the substrate, the dissolved oxygen tension (*DOT*) signal exhibits a negative pulse. This contribution describes and analyzes this *DOT* response in *E. coli* minibioreactor cultivations. In addition to gaining information on culture conditions, a unique response behavior in the *DOT* signal was observed in the analysis. This response appeared only at a dilution ratio per biomass unit higher than a certain threshold. The analysis highlights a plausible relationship between a metabolic adaptation behavior and the newly observed *DOT* signal segment not reported in the literature. A hypothesis that links particular *DOT* segments to specific metabolic states is proposed. The quantitative analysis and mechanistic model simulations support this hypothesis and show the possibility of obtaining cell physiological and growth parameters from the *DOT* signal.

## 1. Introduction

The development of a biopharmaceutical process typically involves three stages. In the first stage, the organisms’ screening and characterization occur. Second, the reaction conditions (e.g., medium and process variables) are optimized. In the last stage, the scale-up to pilot and production scales takes place [1]. During these phases, a high number of cultivation experiments are required [2].

Currently, high throughput technology (HTP) is widely used to accelerate process development [3,4]. To achieve successful HTP cultivations, full automation, miniaturization, and process monitoring and control capabilities are required [3]. Many HTP platforms with miniaturized bioreactors have been commercialized in the last decade. Long et al. [3] provided a nice review on that. The miniaturized bioreactors can be categorized into [5]: (I) the sub-milliliter category, usually called microbioreactors [5,6]; and (II) the 1–10 milliliter category, usually called minibioreactors [5,7]. Bioreactors with a volume of more than 10 mL and usually in the range of 10–100 mL are called small-scale bioreactors [5]. Miniaturized stirred bioreactor systems (MSBRs), sometimes simply called (MBRs) [8,9,10], are designed to resemble the conventional stirred lab-scale bioreactor systems and have been developed as an alternative to shake flasks and microtiter plates (MTPs) [2,11].

The bioprocess development for MBR cultivations has been introduced for different organisms such as *E. coli*, *S. cerevisiae*, and *Bacillus subtilis* [1,5,8,11]. The bacterial species of *E. coli* are suitable candidates for miniaturized systems due to their low susceptibility to shear damage, which allows for higher agitation rates [2]. Normally, *E. coli* cultivations in MBRs rely on intermittent bolus feeding strategies with relatively high frequencies by using automatic pipetting systems for nutrient addition, pH titration, and by taking a limited number of at-line samples. Due to the small culture volume, the collection of offline reference samples is very limited or not possible [2]. MBR reactors usually have different stirring and gassing elements [8,12]. In addition to temperature, pH, and the dissolved oxygen tension, the feeding strategy is known to strongly affect protein expression in *E. coli* [1,13]. Most of the relations describing these effects are based on a completely continuous nutrient addition, which is also the predominant method used in large scale production. In the MBR scale, continuous feeding is hard to realize because of practical reasons [1]. On the other hand, intermittent bolus feeding results in drastic changes in the nutrient concentrations before, during, and after each feeding pulse. Ferenci [14] proposed a concept of “hunger” and “starvation” states, and Vasilakou et al. [15] recently proposed a “feast-famine” concept, both depending on substrate availability. Both contributions reported on the changes in the physiological and metabolic responses. Vasilakou et al. [15] showed that changes happen over short and long periods of time. By considering an immediate glucose addition in a typical *E. coli* culture, the cell consumes all glucose via glycolysis, with one share of the pyruvate being further metabolized via the oxidative pathway, whereas the excess is reduced in the fermentative pathway, also known as the overflow metabolism [15,16,17]. The accumulated acetate can then be consumed oxidatively by the cell. Hence, intermittent feeding leads to frequent changes in metabolic states [1] and transitional oxygen limitation, which can negatively affect cell physiology and growth [1,14]. That is why the oxygen supply is a critical process parameter in aerobic cultivations [18]. Insufficient oxygen transfer to the liquid phase to satisfy the oxygen needs of the cell is a known issue for MBRs [5,19,20,21]. The availability of oxygen in the medium dramatically affects the performance of the cells, leading to drastic changes in the cultivation kinetics [22]. It was noted that overflow can also be detected under continuous feeding conditions by superimposing short pulses to the substrate feed rate (see the work of Akesson et al. [17]).

Dissolved oxygen tension (*DOT*) is a commonly obtained online signal in aerobic cultivations, and measures the oxygen saturation in the liquid phase. In addition to providing information on oxygen availability and saturation in the medium, the signal dynamics contain important information on the cell metabolism. For example, Refs. [17,23,24] showed the possibility of controlling the inhibitory by-product production in *E. coli* by avoiding the anaerobic metabolism using information derived from *DOT* sensors. However, the encoded metabolic activities in the *DOT* signal are ambiguous, and the signal has high and low frequency details; hence, separating the valuable characteristics from the background noise can be difficult. Also, a combination of sensor time delay and high substrate affinity of *E. coli* hinders a clear and direct interpretation of the *DOT* signal [10]. The *DOT* signal is influenced by two opposing components [13]: cell oxygen demand defined by the oxygen uptake rate (*OUR*), and the oxygen transfer to the medium by reactor aeration and stirring systems defined by the oxygen transfer rate (*OTR*). If the stirring and aeration parameters are set at a constant and no control over the dissolved oxygen level in the medium is applied, the metabolic activities described by *OUR* can be revealed in the *DOT* signal. Many contributions reported on the response behavior of the *DOT* signal and the possible relationship between the metabolic activities of *E. coli* and specific responses of the *DOT* signal [16,25,26,27,28,29]. Lin et al. [16] reported on the difference in the slopes of the *DOT* signal with different substrate types. The analysis showed that cells have different oxygen demands and uptake rates for glucose and acetate. The authors hereby did not find any difference if the acetate was introduced from outside the reactor or if it was produced by the cell’s overflow metabolism. After the complete assimilation of all nutrients, the *DOT* signal returns to the saturation value that is mainly governed by the reactor specific oxygen transfer coefficient (*K_la_*), and the oxygen concentration gradient between the gas and liquid phases.

**Figure 1:**
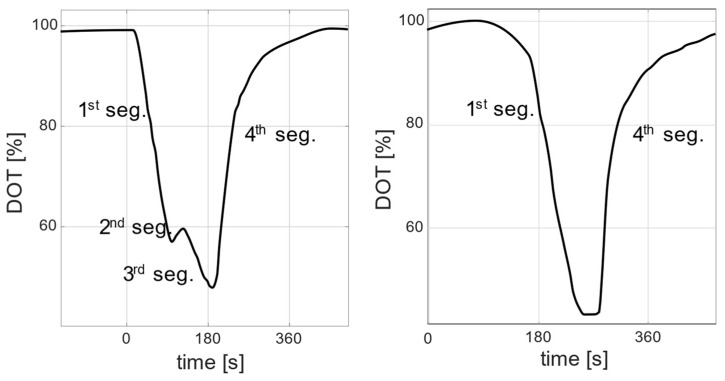
Examples of two *DOT* signals extracted from real experimental data. The signals show different responses to substrate additions: a signal with four segments (**left**) and a signal with two segments (**right**).

In this contribution, in addition to the already reported *DOT* signal behaviors, an additional *DOT* response behavior could be observed. This newly observed response appears as an additional signal segment for a short time under certain conditions. Figure 1 shows two examples of *DOT* pulses in an actual *E. coli* cultivation in a minibioreactor system, in which the following *DOT* segments are distinguished. The first segment occurs after substrate addition and is associated with a direct decline. The second segment occurs after the end of the first segment and is characterized by a slight increase, a flattened curve, or a slight but prolonged decrease. The third segment is aligned with a decline, but with a different slope to the first segment. The fourth segment is aligned with a return to the saturation value.

This contribution presents a systematic experimental study and an analysis of the *DOT* signals recorded in *E. coli* MBR cultivations with intermittent bolus feeding. The feeding plans were designed to have a systematic variation in the feeding frequency and amplitude. The paper’s novelty lies in the detailed analysis of the *DOT* signal and the possibility of retrieving important physical and biological information from the signal dynamics. The hypotheses and quantitative results from the analysis were checked via (I) a comparison to literature values; and (II) mechanistic model simulations. Overall, the analysis promotes a hypothesis on a metabolic adaptation behavior linked to the newly-observed *DOT* segment. The proposed analysis and modelling approaches provide a better understanding of the intermittent bolus-feeding effect on *E. coli* cultivations in MBRs and help address oxygen supply issues. The paper is arranged as follows. Section 2 contains information on the experimental setup and design. Section 3 shows the experimental results and the *DOT* segmentation and correlation results. The inferred hypotheses, quantitative analysis, and simulation results are presented in Section 4. Discussions and future perspectives are presented in Section 5.

## 2. Materials and Methods

### 2.1. Minibioreactor System and Media

For the *E. coli* cultivation experiments, a block of eight minibioreactors (bioREACTOR8; 2mag AG, Munich, Germany) equipped with pH and dissolved oxygen (DO) sensors (Mini-Bioreactors HTBD LG1-PSt3-Hg; PreSens GmbH, Regensburg, Germany) and fluorescence readers (MCR-LG1-v2; PreSens GmbH, Regensburg, Germany) was used. Temperature control and headspace cooling of the bioreactor blocks were achieved by a VersaCool™ Refrigerated Circulating Bath (Thermo Fisher Scientific GmbH, Schwerte, Germany). The gassing and mixing of the culture vessels were provided by the gas-inducing and inductive stirring elements.

An at-line microplate spectrophotometer (SPECTRAmax PLUS384; Molecular Devices Corporation, San Jose, CA, USA) was used for the optical density measurements. A robotic arm (Robotic Manipulator Arm (RoMa); Tecan Trading AG, Männedorf, Switzerland) transferred the samples to a deep freezer storage unit for later HPLC analysis. Glucose and acetate concentrations of the filtered supernatant were analyzed by HPLC (Thermo Fisher, Waltham, MA, USA) with a Supelco gel C-610 H ion exchange column (Sigma-Aldrich, St. Louis, MO, USA) and a refractive index detector (Thermo Fisher, Massachusetts, USA). The mobile phase was 0.1% H_3_PO_4_, with a constant flow rate of 0.5 mL/min at 4 °C. The average sample volume was 300 μL. The headspace of the bioreactors block was cooled to 4 °C to minimize evaporation. The media composition is described in [8].

Experimental runs were conducted with the *E. coli* BL21 strain, carrying an IPTG inducible plasmid encoding for a recombinant protein. The strain variant BL21 is known to produce low amounts of acetate during growth on high glucose concentration media [30].

### 2.2. Experimental Design

To calibrate the lower limit of the DO sensors at 0%, all reactors were gassed with 250 mL/h nitrogen for 20 min, and the stirring speed was set to 2800 rpm. To calibrate the upper limit of the DO sensors at 100%, all reactors were gassed with 250 mL/h air for 20 min.

After calibration, the stirring speed was set to 1900 rpm, and the gassing was set to 62.5 mL/h air for the batch phase, after which the stirrer speed was increased to 2800 rpm. Each experimental run was initiated with 8 mL of medium and 5.7 mg/mL dry cell weight. The batch phase lasted for almost 13 h. After that, the fed-batch started with a bolus feeding of glucose. The concentration of the fed glucose was 600 mg/mL. For two hours at the beginning of the fed-batch, a ramp in the pH from 6.8 to 7.2 was considered to facilitate the induction of the culture. Two hours after the pulsed fed-batch started, the culture was induced with IPTG 76 µL (100 mM). This procedure is part of the fermentation protocol proposed by [8]. Sampling and analysis were started after induction, and five samples were taken throughout the production phase.

**Figure 2:**
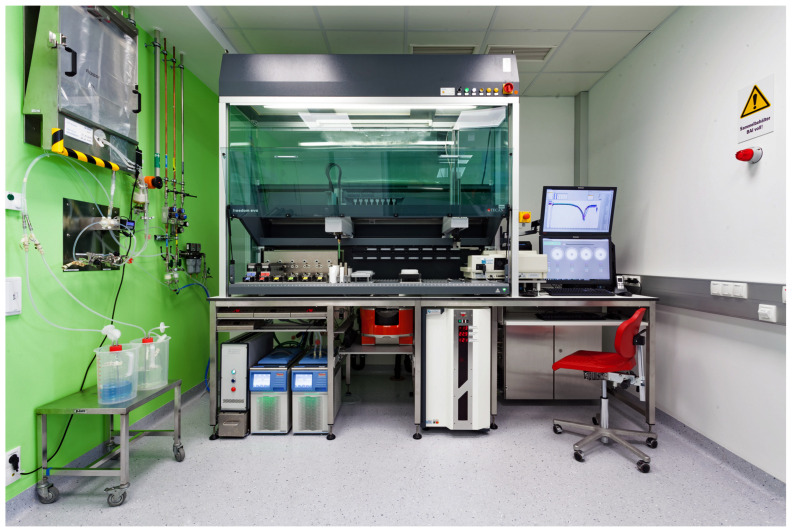
Minibioreactor system (bioREACTOR8; 2mag AG, Munich, Germany), and the experimental setup (Boehringer Ingelheim RCV GmbH & Co KG, Vienna, Austria).

The feeding plan for all reactors is shown in Table 1. In addition to the reference feeding plan [8], which delivers 6.5 µL of glucose solution to the medium (reactor D and E) every 9 min, different feeding plans were also considered. All feeding plans were designed so that the volumes and frequencies of the substrate pulses resulted in the complete consumption of the substrate in between pulses, i.e., it was assumed that no substrate accumulation takes place. In the experiment, the pulses’ amplitude and interval can deviate due to conflicts with other internally scheduled tasks such as sampling or pH titration. More information on the experimental setup and protocols can be found in [8]. A picture of the full experimental system is given in Figure 2.

### 2.3. Computing Platform

All computations for data analysis and numerical modeling were carried out in *MATLAB* R2022a. “*ODE suite*”, primarily *ODE15s,* was used to solve the mechanistic model.

## 3. Experimental Results and Signal Analysis

### 3.1. Experimental Run Results

**Figure 3:**
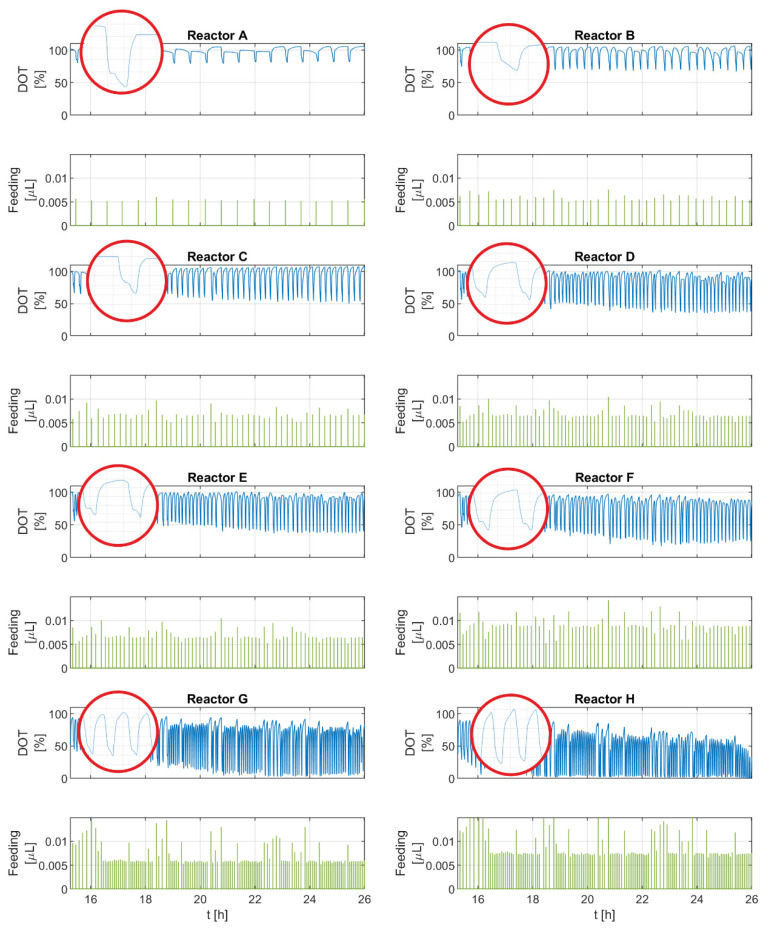
*DOT* signals and intermittent feeding pulses of all experimental runs (reactors A–H).

Figure 3 shows the *DOT* signal and feeding pulses of the eight experimental runs. The *DOT* pulses seem consistent for all runs. Despite some deviations, the glucose feeding pulses generally seem to be equidistant with similar amplitudes for each run, as shown in Table 1.

Overall, the frequency and amplitude of the negative *DOT* pulses correspond with the amount of glucose delivered and the pulse frequency. For runs with a higher glucose addition (F, G, H), the upper and lower boundaries of the *DOT* signal drift downward toward the end of the runs. This trend becomes more pronounced in the experimental runs with the largest feeding volumes, reaching limiting oxygen conditions in G and H. A closer look at the individual *DOT* pulses reveals that some pulses have a different profile than others. These pulses not only show two segments: a straight sharp decline and then a steady return to the saturation value (as commonly described in the literature), but instead they show four segments: first, a sharp decline, which is followed by a transition involving a slight increase, flattened curve, or a slight but prolonged decrease and then another sharp and short decline. After that, a steady return to the saturation value occurs. Both types of pulses are observed in all runs.

The biomass, glucose, and acetate measurement results are shown in Appendix B—Figure A1. The volume changes are shown in Appendix B—Figure A2. Biomass measurements indicate higher biomass in experiments with more frequent nutrient additions and higher nutrient amounts per pulse. The few measured acetate and glucose concentrations are always below 0.5 mg/mL, which indicates no extensive nutrient accumulation, but the existence of acetate indicates a slight overflow metabolism.

### 3.2. DOT Signal Analysis

As observed in the experimental results, each *DOT* pulse is assumed to have four segments, but this is not necessarily the case. It is hypothesized that each segment represents a unique response behavior similar to the responses shown in Figure 1. This assumption is made based on observations derived from the literature and the additional response observed in the experiments. For the sake of the analysis, a segmentation algorithm (described in detail in Appendix A) is built to detect the segments.

**Figure 4:**
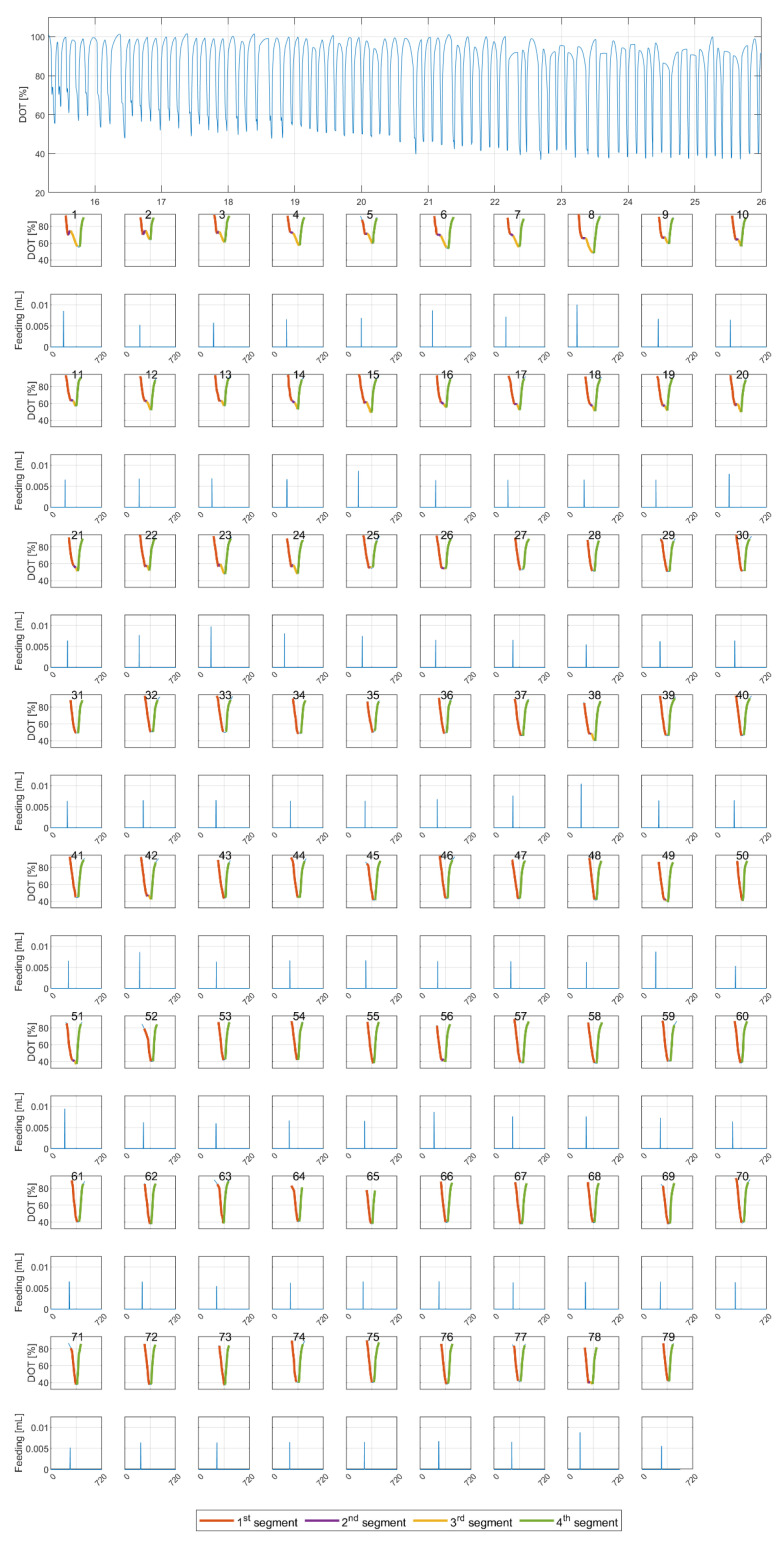
(**Top**) Measured *DOT* signal for reactor E (x-axis is time in hours [h]). (**Bottom**) The results of the segmentation algorithm for all 79 pulses (x-axes represent the individual pulse time in seconds [s]).

The segmentation results are shown for one experimental run (reactor E) in Figure 4. The top subfigure shows the *DOT* raw (interpolated) signal. The subplots below show the segmentation results for each individual pulse. It was noticed that pulses with four segments are generally aligned with feeding pulses with a high amplitude.

After signal segmentation, different segment metrics (descriptive features) were extracted.

**Figure 5:**
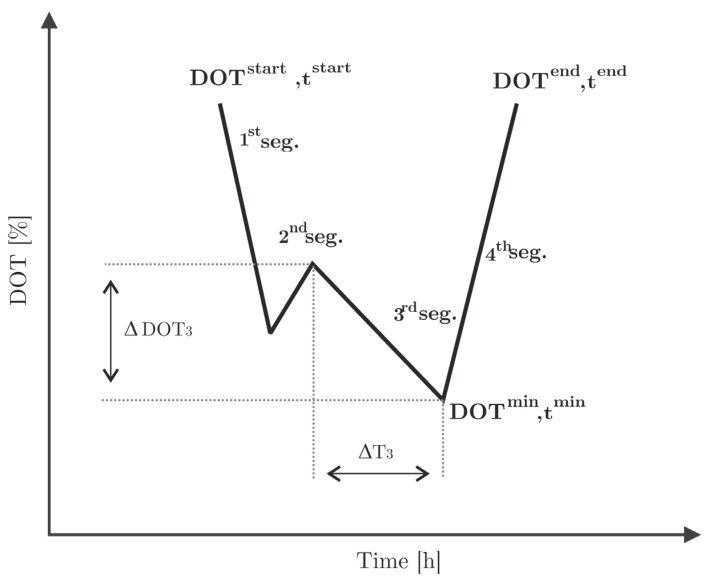
Segmentation of the *DOT* signal and relevant parameters for the quantification of extractable metrics ($\Delta T_{3}$ and $\Delta DOT_{3}$ are given as an example). Each *DOT* pulse is assumed to have four segments, although this is not necessarily the case.

The following metrics are defined for each single (the first, second, third, and fourth) segment and are schematically displayed in Figure 5.

A. Segment time length, defined as

(1) $$\Delta T_{i}=t_{i}^{end}-t_{i}^{start}$$

B. Segment slope, defined as

(2) $$\frac{\Delta DOT_{i}}{\Delta T_{i}}=\frac{DOT_{i}^{end}-DOT_{i}^{start}}{t_{i}^{end}-t_{i}^{start}}$$

C. Segment area, the area under the DOT curve

(3) $$A_{i}=\overset{t^{end}}{\underset{t^{start}}{\int }}DOT_{i}\;dt$$

These metrics were calculated for every pulse in the eight experiments, and were later correlated with process parameters such as the dilution ratio or biomass and acetate measurements. The correlations are qualified by the $R^{2}$ goodness of fit parameter and the r Pearson correlation coefficient. Table 2 gives an overview of all metrics considered, and corresponding results are given in Appendix D. In the following paragraphs, only meaningful results with sufficiently large $R^{2}$ and r were selected and discussed in detail.

Similarly, the area of the *OTR* is calculated once the $K_{La}$ value is known. Based on this area, the overall oxygen mass transferred to the medium is calculated.

**Metric A: Segment Time Length**

**Figure 6:**
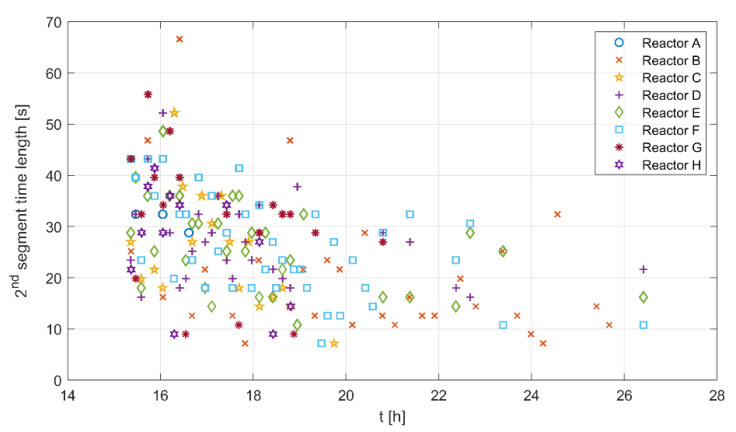
Second segment time length for all experimental runs along the cultivation time course.

A segment length gives some information on how long a certain metabolic state lasts. Figure 6 shows the time length of the second segment for all experimental runs along the time course of the cultivations. The time length seems to be high for all runs at the beginning and lower toward the end of the cultivation. The second segment does not appear for all glucose pulses, as shown in Figure 4, and here the time becomes shorter toward the end of the cultivation. The mean of the reported values is 31 s, with a 6.5–55.5 s range for two standard deviations.

**Figure 7:**
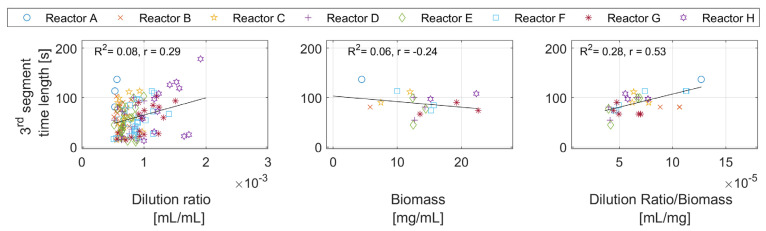
Time length of the third segment against the dilution ratio, the biomass concentration, and the dilution ratio per biomass concentration.

Figure 7 shows the third segment time length against the dilution ratio, biomass concentration, and dilution per biomass unit. Based on the overall hypothesis, the third segment is linked to the oxidation of the formed acetate. The relationships between the segment time length and the feed amount and biomass concentration could provide information on the formed acetate amounts. The points for the biomass were calculated for the pulses in the close vicinity of the samples where the biomass concentration change is neglectable.

The figure shows almost no correlation between the third segment time length and the dilution ratio, and a weak relationship with biomass. This is because the effects of the biomass and the dilution ratio are alternately overlooked from many points in both figures. For example, a high dilution ratio with a large biomass concentration results in the same time duration as a low dilution ratio with a low biomass concentration. However, there seems to be a correlation with the dilution ratio per biomass unit.

Results for the first, second, and fourth segments are given in Appendix D—Figure A3.

**Metric B: The Slope of the Segments**

**Figure 8:**
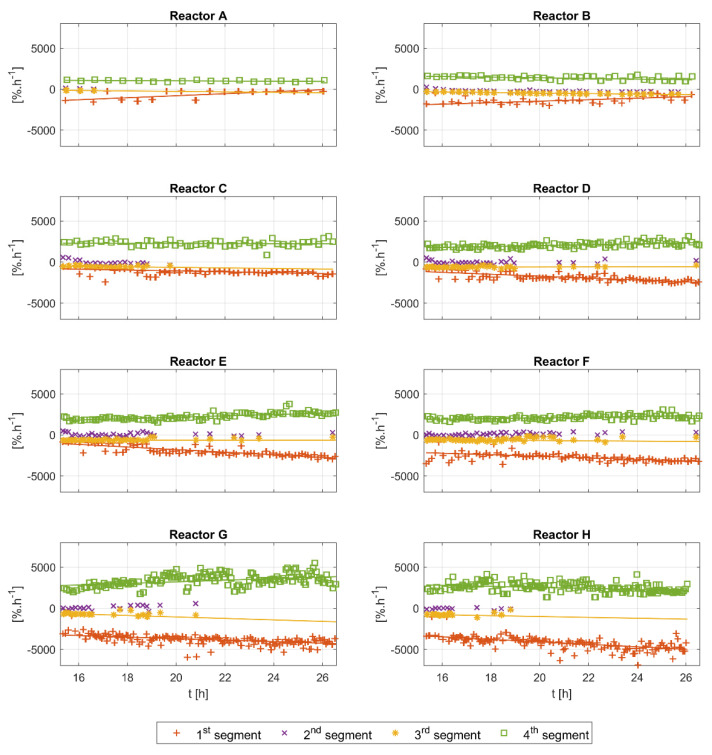
Slope analysis of the first, second, third, and fourth segments of all experimental runs (reactors A–H). The slopes of the segments tend to be relatively constant along the time course of each cultivation. The second and third segments do not appear for each *DOT* pulse.

The analysis of the slopes of the detected segments is shown in Figure 8. In general, the slope contains information on the overall speed of the reactions, and therefore information on how rapidly glucose and acetate are consumed, as well as the oxygen transfer rate of the reactor system. All segments’ slopes appear to have a relatively similar and constant trend in all runs. The slopes of the first and third segments show negative values with a visible difference between them. The slope of the fourth segment is always positive. For the second segment, positive values are detected at the beginning of the runs, and then the values become lower and closer to zero, or slightly negative. Again, the second and third segments do not appear for all glucose pulses. The slopes of the first, third, and fourth segments drift slightly towards the end of the cultivations. The Bartlett statistical tests for all runs show high values ($x^{2}\approx \left[1150,\;2600\right]$ with p-value=0), and the ANOVA test also shows high values for all runs ($F\approx \left[1700,\;97,000\right]$ with p-value=0), suggesting a significant difference between the slopes of the different segments.

**Metric C: The Area of the Pulse**

**Figure 9:**
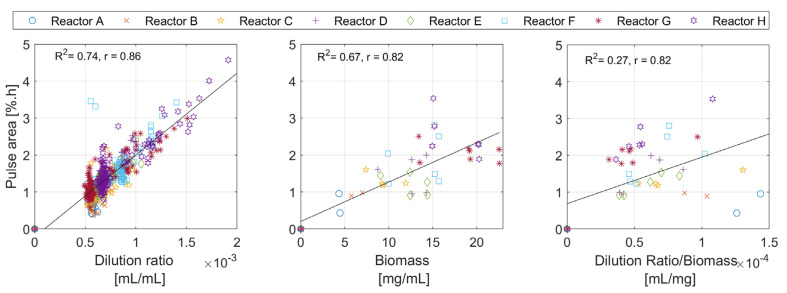
The analysis of the individual *DOT* pulses areas plotted against the dilution ratio, the biomass concentration, and the dilution ratio per biomass concentration.

The area metric can only be determined for the overall pulse, and it contains information on the overall amount of oxidatively consumed nutrients. Figure 9 therefore suggests a possible correlation between the area of the *DOT* pulse with the dilution ratio and the biomass concentration. The results in the figure (right) are only plotted in the neighborhood of the biomass samples, where the biomass concentration change is neglectable.

## 4. Retrieval of Physiological Information from *DOT* Signal Segments

### 4.1. Physiological Analysis of the Segments

#### 4.1.1. Hypothesis on the Physiological Meaning of the Segments

From the visual inspection of the figures in Section 3.2, the following hypotheses on the segments can be made:

The first segment starts after adding the glucose to the medium with a short delay of roughly 2–4 [s]. The slope of this segment is always negative and smaller than the third segment slope. Cells in this segment consume the available glucose by glycolysis and the resulting pyruvate is further oxidized to CO_2,_ and some excess is reduced to acetate in the overflow regime. As expected, the amount of accumulated acetate in the medium depends on the volume of the glucose added, the overall biomass concentration, and the oxygen availability.

In the second segment, the segment’s length and slope differ between the experiments and along each experiment. This segment can be noticed because of the increase to a higher *DOT* value; a flat, or a very slow decrease of the *DOT* signal, which indicates a transition phase from the first segment to the third segment; and a potential adaption time from glucose to full acetate oxidation capacity. The second segment appears at a dilution ratio per biomass unit higher than a certain threshold.

In the third segment, the slope is always negative but is less steep than the first segment slope. The cells are assumed to oxidize the accumulated acetate at their full capacity. It is possible that the acetate is already partly oxidized during the second segment.

The fourth segment starts when the *DOT* pulse reaches the minimal value and ends when the *DOT* reaches its starting point. This segment features a return to higher *DOT* values, mostly (but not necessarily) to the saturation value. The metabolism in the whole fourth segment is assumed to be inactive, and the increase is mainly driven by the oxygen transfer rate of the reactor system.

#### 4.1.2. Quantitative Analysis

**Figure 10:**
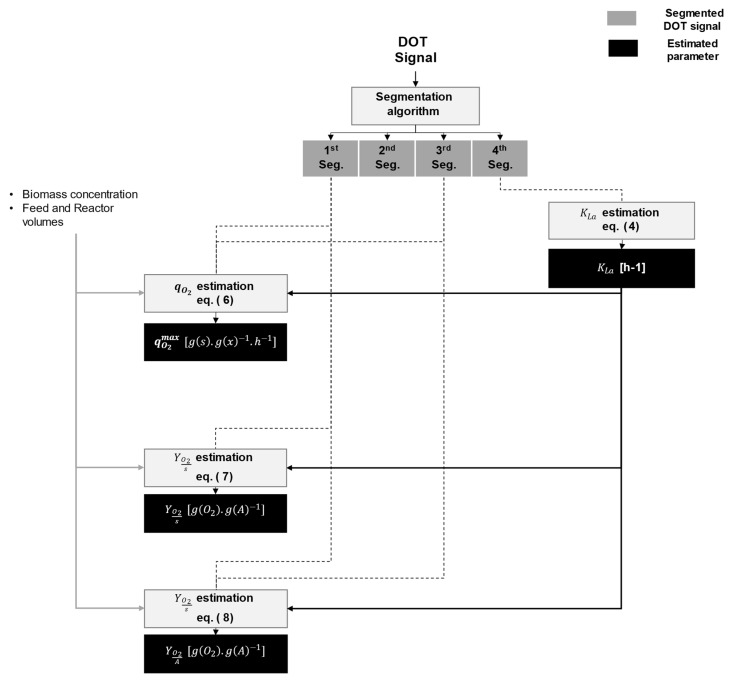
Parameter estimation workflow using *DOT* signal segmentation. The workflow can be used for the estimation of the overflow condition parameters in *E. coli* using biomass samples, provided that the feeding and reactor working volumes are known.

Figure 10 shows a proposed workflow to extract physiological information using the segments and metrics described in Section 3.2 and the hypotheses in Section 4.1.1. Based on the four identified segments, the following cell physiological information can be retrieved: the maximum biomass specific oxygen uptake rate ($q_{O_{2}}^{max}$), the oxygen to substrate yield ($Y_{O2/S}$), the oxygen to acetate yield ($Y_{O2/A}$), and the reactor-specific oxygen transfer rate ($K_{la}$). See Appendix C and Table A2 for the mechanistic model, the nomenclature, and the description of the parameters.

For example, the $K_{La}$ value can be calculated with the help of the fourth segment’s time length using Equation (4), which is the analytical solution of $dDOT/dt=\left(DOT*-DOT\right)\cdot K_{La}$:

(4) $$K_{La}=-\frac{\log\left(\frac{DOT*-DOT^{end}}{DOT*-DOT^{min}}\right)}{\Delta T_{4}}\;\;$$

where DOT* is the signal at saturation, and $\Delta T_{4}$ is the time length of the fourth segment. To account for the sensor delay in the signal, the actual dissolved oxygen signal (*DOT*) was obtained from the measured dissolved oxygen signal ($DOT_{m}$), following:

(5) $$DOT=\tau \cdot \frac{dDOT_{m}}{dt}+DOT_{m}$$

The slope of the first segment can give information on the actual specific glucose uptake rate $q_{s\;\left(ox\right)}$. In the first segment, the cell is assumed to consume glucose at full capacity, therefore $q_{s\;\left(ox\right)}=q_{s\left(ox\right)}^{critical}=q_{O_{2}}/Y_{O_{2}/s}$.

In the third segment, the cell is assumed to oxidate only to acetate, where $q_{A\;\left(ox\right)}=q_{A\left(ox\right)}^{critical}=q_{O_{2}}/Y_{O_{2}/A}$.

In the first segment, the specific oxygen uptake rate $q_{O_{2}}$ for a *DOT* pulse is calculated as:

(6) $$q_{O_{2}}=\frac{{\left.\frac{dDOT}{dt}\right|}_{\left[\Delta T_{1}\right]}+\left(DOT*-DOT_{1}^{end}\right)\cdot K_{La}}{H\cdot C_{x}}$$

where H is the Henry derived constant. Note that Equation (6) was only used for *DOT* pulses in the neighborhood of a biomass sample, i.e., where concentrations $C_{x}$ were available. $q_{O_{2}}$ can be similarly calculated from the third segment.

The cell physiological parameter $q_{O_{2}}^{max}$ can thus be calculated under the assumption that the instantaneous glucose addition causes a maximum oxygen uptake rate, at least at the beginning, simply as $q_{O_{2}}^{max}=MAX(q_{O_{2}})$. The previous assumption holds true for the third segment only if the accumulated acetate concentration is high enough to cause maximal uptake in the cell. Therefore, it is better to calculate $q_{O_{2}}^{max}$ using the first segment.

The amount of oxygen needed to oxidize a certain amount of glucose is determined by the stoichiometric yield coefficient $Y_{O2/S}$. This value can be either calculated from the stoichiometric matrix or estimated empirically as a model parameter. However, in a *DOT* pulse with only first and fourth segments and equal start and end values, the amount of oxygen delivered to the cell is known: $O_{2}\;mass\;\left[mg(O_{2}\right)]=V\;\cdot \int OUR\cdot dt=V\cdot \int OTR\cdot dt=V\int \left(DOT*-DOT\right)\cdot K_{La}\;dt$. The amount of glucose delivered to the cell during this time window is known; therefore, the yield can be calculated using the area of the segments as:

(7) $$Y_{O2/S}=\frac{V\;\cdot \;\int_{t^{start}}^{t^{end}\;}\left(DOT*-DOT\right)\cdot K_{La}\;dt}{F_{s}\;\cdot \;C_{s,in}\;}$$

where V is the reactor working volume, and $K_{La}$ is the estimated value from Equation (4).

Similarly, the amount of oxygen needed to oxidize a certain amount of acetate is usually determined by the stoichiometric yield coefficient $Y_{O2/A}$. The amount of accumulated acetate can be determined from the glucose flux that exceeds the maximum oxidative capacity. This can be written as: $Acetate\;mass\;\left[mg\left(A\right)\right]=\left(F_{s}\;\cdot \;C_{s,in}-\int q_{s\;\left(ox\right)}^{max}\cdot dt\;\cdot C_{x}\right)\cdot \;Y_{A/S}$.

The amount of oxygen that goes to oxidize the acetate can be extracted by integrating the oxygen uptake rate along the third segment and substituting for the *DOT* difference between $t_{3}^{start}$ and $t_{3}^{end}$. This can be easily calculated by extrapolating *DOT* from $t_{3}^{end}$ to the time point $t_{3}^{ext}$, where the *DOT* value equals the *DOT* value at $t_{3}^{start}$. Using the area of the first and third segment, the yield is therefore obtained as:

(8) $$Y_{O2/A}=\frac{V\;\cdot \;\int_{t_{3}^{start}}^{{\;t}_{3}^{ext}}\left(DOT*-DOT\right)\cdot K_{La}\cdot dt}{\left(F_{s}\;\cdot \;C_{s,in}-\int_{t_{1}^{start}}^{t_{1}^{end}}q_{s\;\left(ox\right)}^{max}\cdot dt\cdot C_{x}\right)\cdot \;Y_{A/S}}$$

The numerical results obtained from the rigorous analysis of the *DOT* signal segments are listed in Table 3, indicating the range within one standard deviation.

### 4.2. Simulation Example to Reconstruct the DOT Response Behavior

In order to further analyze the relevance of the obtained physiological parameters and to investigate the hypothesis for the second segment, a well-accepted growth model describing the overflow metabolism in *E. coli* was considered [31]. It is a piece-wise continuous model, with specific sub-models for each metabolic state. Model switches are numerically implemented using the Event Driven Method (EDM), see, for example, [32] for details. This approach seeks an accurate location of the metabolic events and is distinctly different from other approaches focusing on formulating a continuous metabolic transition, e.g., [33]. The adopted piecewise modeling approach can help to acquire accurate results [32], especially when the changes happen in a short timescale.

The actual dissolved oxygen signal (DOT) is measured with a first-order delay $\tau =36\;\left[s\right]$ caused by the response time of the sensor, therefore an additional equation for the measured dissolved oxygen signal ($DOT_{m}$) is also considered. The actual dissolved oxygen equation reads:

(9) $$\frac{dDOT}{dt}=OUR-OTR=\left(DOT*-DOT\right)\cdot K_{La}-\left(Y_{O2/S}\cdot q_{s\;\left(ox\right)}+Y_{O2/A}\cdot q_{A\;\left(ox\right)}\right)\cdot H\cdot C_{x}$$

After considering the probe response time, the measured dissolved oxygen reads:

(10) $$\frac{dDOT_{m}}{dt}=\frac{1}{\tau }.\left(DOT-DOT_{m}\right)$$

More on the model and the nomenclature is found in Appendix C.

Following the hypotheses made in Section 4.1.1, the following metabolic states are considered:

Metabolic state I: Glucose oxidation with an overflow metabolism. The cells consume glucose at the maximum oxidative uptake rate and the excess glucose is reduced after glycolysis to acetate. This state is active during the first segment.

Metabolic state II: The transition from glucose to acetate oxidation. The cell metabolism is limited by glucose depletion and the inability to immediately oxidize the formed acetate at the full capacity. This state is always active after the end of the overflow metabolism (metabolic state I) when the acetate accumulation exceeds a certain threshold (assumed to be 0.1 [mg/mL], similar to the values found in the literature [16,34]).

Metabolic state III: acetate oxidation. The acetate is exclusively oxidized. This state is active during the third segment.

Metabolic state IV: static state. No active metabolic activities are detected. This state is active the whole time, except when there is a glucose pulse. It is also active during the fourth segment.

**Transition from Glucose to Acetate Oxidation as a Model Extension**

In the transition between glucose and acetate oxidation, all model rates q including $q_{A\;\left(ox\right)}$ and $q_{s\;\left(ox\right)}$ are set to reduced values $q_{adap}$ by a reduction factor $R\left(t\right)$. A reduction factor of $R\left(t\right)=100\%$ means that the cell stops fully to uptake the substrates. For this contribution, a complete reduction of metabolic activities is assumed, although in reality the cell maintenance is still active. In this simplified version, model rates can then be written as:

(11) $$q_{adap}=q\cdot \left(1-R\right)$$

The biomass change during this short time window can be negligible. The relevant model parameters are taken from the analysis results made earlier in Table 3 (for reactor E). The rest of the model parameters are listed in Appendix C—Table A2.

**Constant Adaptation Time**

**Figure 11:**
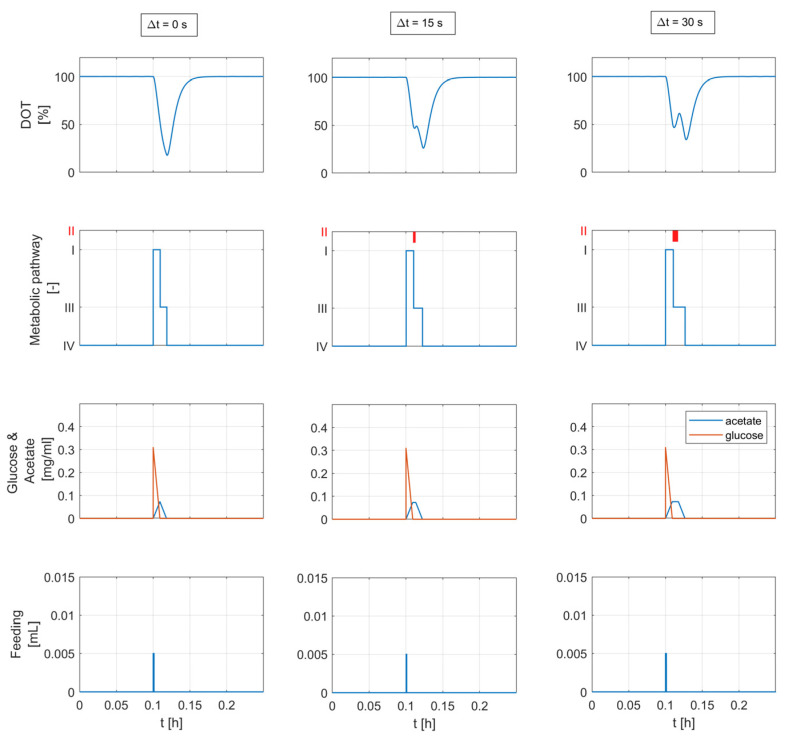
Simulation results considering a single feed injection and three different values of the adaptation time. The relevant concentrations and model parameters are calculated from the *DOT* signal analysis in Section 4.1.2 and are shown in Table 3 (experimental run E). The plots in the second row show the activity of the metabolic states: (**I**) overflow metabolism, (**II**) adaptation state: metabolism is paused, (**III**) acetate oxidation, and (**IV**) static: no active substrate metabolism. The consideration of the adaptation state (∆t > 0) yields the second segment in the predicted *DOT* signal.

The time length of the adaptation state Δt is set to a constant value within the experimentally observed range [6.5, 55.5] seconds, see Figure 6. Figure 11 shows the simulation results for different adaptation times (Δt=0, Δt=15, Δt=30 s) considering a substrate pulse of 5 μL.

**Considering Other Factors Affecting the Adaptation Time**

The accumulated acetate and biomass concentrations adversely influence the time length of the adaptation state Δt; therefore, it is proposed to be defined as:

(12) $$\Delta t=t_{adap}^{max}\left(\frac{\;C_{A}}{C_{A}^{max}}\cdot \frac{C_{x}^{max}}{\;C_{x}}\right)$$

**Figure A1:**
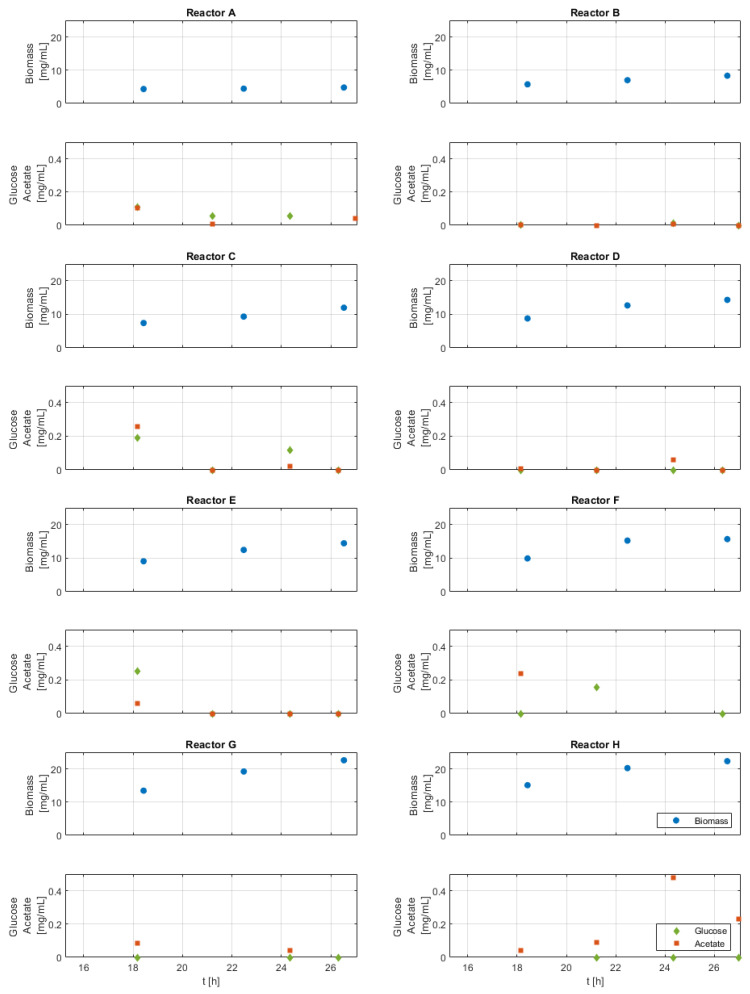
Biomass, glucose, and acetate concentrations of each run (reactors A–H) in the time window of the analysis. Due to working volume limitation, only a limited number of off-line/at-line samples are possible.

$C_{A}^{max}=0.5\;\mathrm{mg}/\mathrm{mL}$ and $C_{x}^{max}=25\;\mathrm{mg}/\mathrm{mL}$ are the maximum acetate and biomass concentrations, respectively, and $t_{adap}^{max}=60\;s$ is the maximum adaptation time that is observed in the analysis. These values have been taken from the analysis results in Section 3 (see Figure 6 and Figure A1).

**Figure 12:**
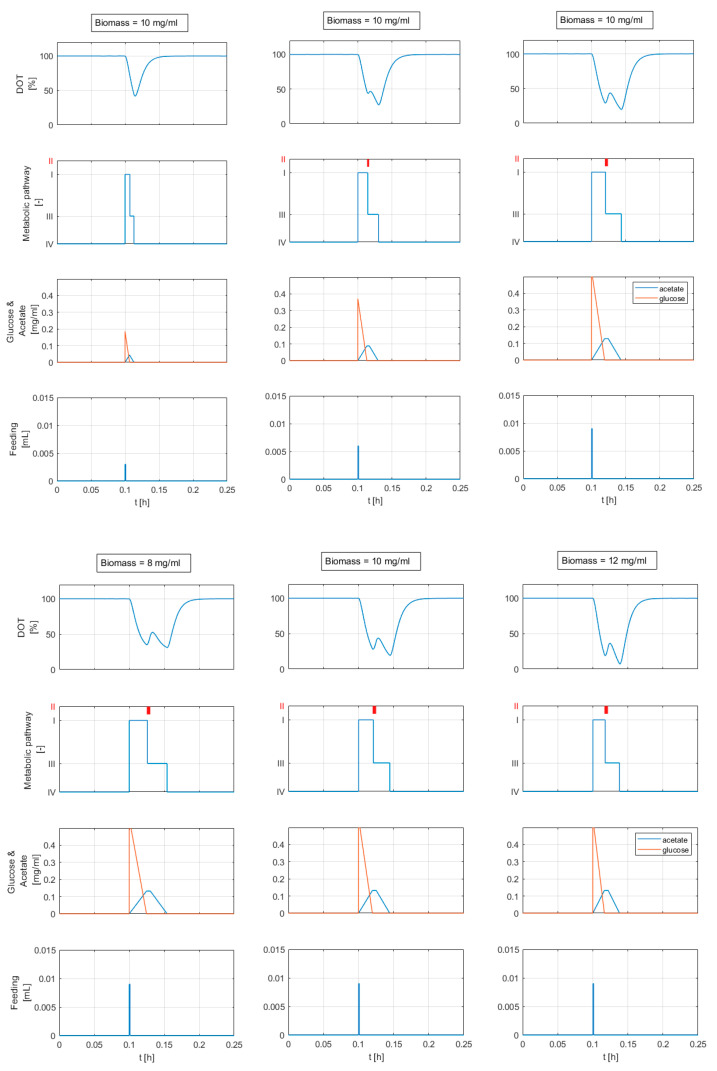
Simulation results for different feed volumes and a constant biomass concentration (**top**), and for different biomass concentrations and a constant feeding pulse volume (**bottom**). The relevant concentrations and model parameters are calculated from *DOT* signal analysis in Section 4.1.2 and are shown in Table 3 (experimental run E). The plots in the second row show the activity of the metabolic states: (**I**) overflow metabolism, (**II**) adaptation state: metabolism is paused, (**III**) acetate oxidation, and (**IV**) static: no active substrate metabolism. If the glucose feed volume exceeds a certain threshold (5 μL), the second segment in the *DOT* signal appears.

Figure 12 (top) shows the simulation result for three different glucose feed volumes (3, 6 and 9 μL) and a constant biomass concentration of 10 mg/mL. The results in Figure 12 (bottom) are computed for increasing biomass concentrations (8, 10, and 12 mg/mL) and the same glucose addition of 9 μL.

In both cases (constant and variable adaptation times with Δt>0), the *DOT* signals exhibit a response behavior similar to the behavior reported in Figure 1. The computed *DOT* signals show four segments: a decline, a small increase, another decline with a different slope, and, finally, a return to the saturation value. The second and third segments appear when the glucose feed volume exceeds 5 μL. The time length and height of the second segment are related to the added glucose volume and biomass concentration (see Figure 12). A glucose feed below 5 μL results in a *DOT* pulse with two segments: a straight decline followed by a return to the saturation value.

## 5. Discussion

### 5.1. Hypothesis Verification and Quantitative Analysis

The hypothesis with regard to the relationships between the metabolic activities and the existence of the *DOT* signal segments is supported by the results of the quantitative analysis and the mechanistic model simulations. Additionally, the following observations are made:

First segment: the observed delay time (2–4 s), after which the cell starts to actively metabolize glucose, seems within the range of $\tau_{4}$, as reported by [25]. The authors referred to this delay as the “light-off phenomenon”. The assumption about cells metabolizing the glucose in the overflow metabolism is in alignment with findings in the literature [15,16]. Since the pulse injection time is very short (around 1 s), a sudden increase in glucose concentration in the medium is expected. This triggers the overflow metabolism if the maximum specific glucose uptake rate $q_{s\;}^{max}$ is assumed to be greater than the maximum oxidative capacity $q_{s\left(ox\right)\;}^{critical}=q_{O_{2}}/Y_{O_{2}/s}$ of the cell. The relatively low $q_{O_{2}}^{max}$ value in Table 3 supports this assumption.

Second segment: the change of the slope of this segment seems to be positively correlated with the amount of accumulated acetate and negatively correlated with the biomass concentration. This segment appears only after a certain dilution ratio per unit of biomass unit value. From our analysis, we would expect the dilution ratio per biomass unit threshold to be approximately 0.5 to 0.8 µL(glucose)/mg(biomass). However, a thorough verification of the factors affecting the time length of this segment was not possible due to the due to the sparsity and lack of a sufficient amount of biomass and acetate samples.

Third segment: this segment appears only after the second segment. Figure 7 shows a likely positive correlation between the segment length and the dilution ratio per biomass unit. A plausible explanation is that with a high enough dilution ratio per biomass concentration, the acetate production under the overflow metabolism in the first segment is triggered. In the third segment, the cells consume the accumulated acetate. The time required for that is correlated with the amount of acetate produced, and by that, the time length is correlated with the dilution ratio per biomass unit.

Figure A1 shows no acetate accumulation in the neighborhood of the *DOT* pulses. This further supports the notion of a transient production of acetate in the first segment and the transient and full consumption of acetate in the third segment. Additionally, for the third segment, there is assumed to be a negligible to no glucose concentration in the medium for this time window. Figure A1 shows no considerable glucose concentration for all runs. However, due to the sparsity and the lack of sufficient glucose and acetate samples, this cannot be thoroughly verified.

Fourth segment: the assumption with regard to the inactive metabolism in this segment matches the findings in the literature.

The pulse area analysis in Figure 9 shows relatively linear trends, suggesting a possible relationship with two factors: the amount of glucose added to the medium, and the biomass concentration. The exact relationship between the areas and these factors is difficult to estimate due to the low number of biomass samples. The area of the pulses is directly linked to the amount of oxygen deposited in the medium along the time span of the DOT pulse. Equation (7) shows one possible mathematical description of this observation. The absolute amount of glucose added is known, and the absolute amount of oxygen consumed per unit of biomass concentration can be calculated by integrating the oxygen uptake rate (*OUR*) over the time window of the pulse; hence, the cell oxygen to glucose yield $Y_{O2/S}$ can be calculated. Once $Y_{O2/S}$ is calculated, the oxygen to acetate yield $Y_{O2/A}$ can also be calculated with the help of glucose to acetate yield $Y_{A/S}$, as described in Equation (8).

Table 4 summarizes the literature values relevant to the analysis of this work.

The value of OUR and $K_{La}$ obtained from the *DOT* signal analysis (Table 3) are comparable to the literature values of experiments with a similar volume and biomass concentration. $q_{o}$, the oxygen specific uptake rate value, seems slightly lower than the value reported by [18].

The $Y_{O2/S}$ and $Y_{O2/A}$ values are lower than the values reported in the literature. Lin et al. [16] reported values of almost 1 g/g for both yields. Anane et al. [33] reported yields of 1.56 and 0.54 g/g, and they later reported yields of 1.08 and 1.2 g/g for $Y_{O2/S}$ and $Y_{O2/A}$ consecutively [40]. The first two contributions show modelling results for a lab scale reactor, and the last one shows the reported results for the minibioreactor scale. However, these contributions did not indicate the corresponding $K_{La}$ value.

A common challenge when estimating model parameters is to determine the $K_{La}$ value that set the delicate balance between the two components of the *DOT* signal: the oxygen uptake rate and the oxygen transfer rate. These components are mainly influenced by the values of the parameters $K_{La}$ on one side and $Y_{O2/S}$ and $Y_{O2/A}$ on the other side (given $q_{O_{2}}^{max}$ is estimated and has a fixed value). The positive correlation between the parameters means that high yield coefficient values imply high $K_{La}$ values, and vice versa. Therefore, a high $K_{La}$ is expected for the previous contributions. The quantitative analysis results in Table 3 show relatively comparable $K_{La}$ values that match the literature findings in Table 4.

By estimating the parameters $\;q_{O_{2}}^{max}$ and $Y_{O2/S}$, the overflow switching condition $q_{s\left(ox\right)\;}^{critical}=q_{O_{2}}/Y_{O_{2}/s}$ is identified. The oxygen affinity constant $K_{O}$ appears to be an insensitive parameter in our analysis.

The slope of the fourth segment is directly linked to the volumetric mass transfer coefficient $K_{La}$. The almost constant values of the fourth slope can be seen for all runs in Figure 8, indicating an almost constant $K_{La}$ value along the time course of each run. However, a comparison of the slope of the fourth segment between the runs shows a negative correlation with more feeding. The relevant process variables, such as stirring speed and aeration rate, are the same and are kept constant for all experimental runs. Therefore, the observed drift might be explained by changes in the medium’s characteristics. With more feeding, the viscosity of the medium changes mainly because of the higher biomass concentration.

It is worthy of note that the highest estimated $K_{La}$ values in Table 3 are reported for runs D and E. These runs are duplicates of the standard feeding plan reported in [8]. Higher feeding plans (i.e., runs F, G and H) seem to result in higher viscosity, resulting in a lower delivery of oxygen from the gas to the liquid. Lower feeding plans (i.e., runs A, B and C) seem to result in lower working volumes, which might negatively affect the oxygen transfer rate delivery.

The slopes of the first and third segments in Figure 8 have consistent values within each run, but show small differences between the runs. This can be explained by the changes in the uptake rates in the new cell generations caused by the intermittent feeding [15]. The slopes of these segments can be linked directly to the oxygen uptake rate $q_{O_{2}}$. However, given an almost instantaneous addition of the substrate, a maximum and constant value of the oxygen uptake rate can be expected for most of the time in the first segment. For the third segment, a similarly constant value of $q_{O_{2}}$ is probable if enough acetate accumulates. If that is the case, then the difference in the slopes of the first and third segments can be explained by the difference in the oxygen to glucose yield $Y_{O2/S}$ and the oxygen to acetate yield $Y_{O2/A}$. However, the lack of enough acetate samples hinders a reliable validation of this hypothesis, but the quantitative analysis provided in Section 4.1.2, does suggest a difference in the values of the $Y_{O2/S}$ and $Y_{O2/A}$.

**Figure A4:**
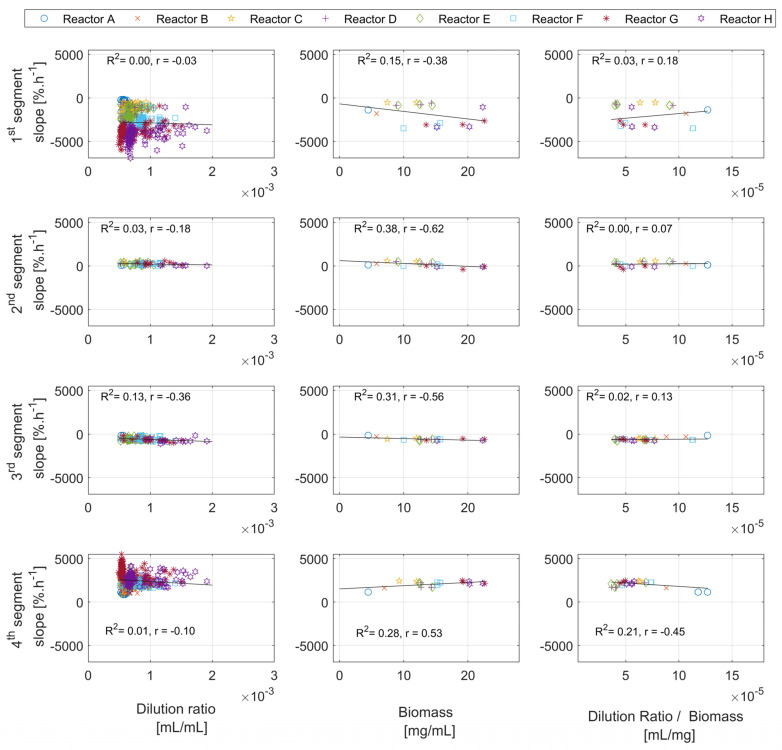
Slope of the segments against the dilution ratio, biomass concentration, and the dilution ratio per biomass concentration.

In all experimental runs, the second segment slope starts with positive values for the first few hours, then becomes close to zero, and in some instances even becomes slightly negative. This indicates a continuous slope change during cultivations, from a positive to a negative slope. The range of slopes in which the second segment is detected is defined by the segmentation algorithm, in which tuning parameters are dynamically estimated during the training of the algorithm (see Appendix A for the details). The biomass concentration (not visible in Figure A1) and the working volume of all reactors have similar values at the beginning of the analyzed time window, and therefore the high slope values in the first couple of hours can be explained exclusively by the large feeding pulses. A visual inspection of Figure 4 reveals that the existence of the second segment is usually linked to feeding pulses with high amplitudes. The correlation analysis for the second segment’s time length and the biomass concentration reveals a negative correlation (the results shown in Appendix D, Figure A4).

#### Newly Observed *DOT* Response Behavior

Figure 6 shows the detected time length of the second segment. The figure depicts values in the range of 6.5 to 55.5 s, with a mean value of approximately 31 s.

Refs. [25,26] reported on the metabolic response of *E. coli* to glucose pulses by using a bioluminescent reporter strain (DPD2085, yciG::luxCDABE) that allows for an online monitoring of the changing metabolism. Their observations showed that *E. coli* can switch from overflow to acetate oxidation “rapidly”, and this switch is usually aligned with an overshoot in the bioluminescence, with a peak lasting for almost a minute.

This metabolic change could happen because of cell stress, or when part of the cell population switches while the rest do not, or as a mix of both factors.

Refs. [27,28] applied nuclear magnetic resonance techniques to monitor the metabolic switches in *E. coli*. Their observation showed a rapid induction of “acs”, the gene responsible for acetate synthase after the metabolic switch (from overflow to acetate oxidation). An overlapping time window between acetate consumption and acetate production might have an effect on cell metabolism in the time window around the switch in which a co-utilization of acetate and glucose occurs. Furthermore, the authors reported a drop in the growth rate directly after the switch.

Ref. [29] used Isotope Dilution Mass Spectrometry (IDMS) to analyze the metabolic changes after a glucose pulse at a timescale measured in seconds. Interestingly, *E. coli* can store relevant amounts of carbon to be used after the overflow in a period of tens of seconds.

Ref. [16] also showed a pulse-based method for the determination of the maximum uptake capacities for glucose and oxygen in glucose limited cultivations. Their observations showed that acetate is formed after a glucose pulse. However, the redirection of the acetate flow from production to consumption takes some time. The authors did not report on a specific time duration. In their contribution, the sampling time interval of the *DOT* signal was 5 s, and a change in the *DOT* signal similar to the second segment presented in this contribution was shown for a few sampling points. However, this is neither highlighted nor discussed. The authors also report on the increase in the $q_{o}$ rate after the glucose pulses. This was attributed to the “uncoupling effect” (inhibition effect of the acetate), although the added acetate concentration was low.

The previous observations suggest a metabolic switching time similar to the time range reported in Figure 6, and provide possible explanations of the metabolic changes in this time window. Hence, incorporating an “adaptation state” in the model, which represents a reduction in the metabolic activities after switching from the overflow metabolism, seems feasible.

The results of the mechanistic modeling analysis in Figure 12 show that considering an adaptation state (Metabolic state II) in which the metabolism is paused for a short time results in *DOT* signal changes similar to the second segments seen in the raw data. The simulations also show that the second segment appears clearly and becomes more pronounced with larger feeding volumes after a certain threshold. A larger feeding volume means that cells need more time to fully consume the glucose added to the medium. As the cell is already working at its maximum uptake rate, the excess sugar is metabolized anaerobically, and acetate accumulates in the medium. The second segment becomes more pronounced as a result of the prolonged adaptation state caused by higher acetate accumulation.

### 5.2. Industrial Relevance

The minibioreactor systems are increasingly seen as useful tools in the pharmaceutical and bioprocessing industries for purposes such as strain screening and experimental design. They do not inherit some larger scale issues such as inhomogeneity, mixing, and aeration difficulties, and they offer an economically viable method of cutting costs. However, scaling experiments up/down from/to the milliliter scale remains a challenging issue. Anane et al. [40] recently reported on this, and they showed deviations in parameter values compared to their reference cultivation and reported on an increased amount of some amino acids (particularly norvaline) when bolus feeding was used. Our observations further indicate that frequent metabolic switches could have a negative impact on the key parameters of the cell.

The hypothesized adaptation phenomenon that repeatedly happens in minibioreactor systems with intermittent bolus feeding seems to cause frequent cell stress. The relatively low values of the estimated parameters (e.g., $q_{O_{2}}^{max}$,$Y_{O2/S}$ and $Y_{O2/A}$) and the general tendency towards lower values for an increased feeding frequency (reported in Table 3) underlines the negative impact on cell metabolism.

Additionally, in larger reactor scales, the inhomogeneities in the medium can trigger a similar behavior of metabolic switching in some local regions in the reactor [15]. The proposed analysis, by quantifying the metabolic adaptation time, can be used as a strain selector to choose strains that can better endure these effects.

### 5.3. Future Outlook

Further in vitro investigations of the physiology behind the adaptation state in minibioreactor systems is needed to reveal more about this phenomenon on a genetic, proteomic, and metabolic level.

Future experimental plans to overcome the practical limitations of the used minibioreactor systems can help provide a more detailed analysis and provide more information on the cell status. For example, additional information on the maximum cell substrates uptake rates can be obtained by sampling immediately before and after the glucose pulse.

Further model-based analysis to calculate the sensitivities of all model parameters to the *DOT* signal, and the degree of metabolic reduction in the time window of the adaptation state could help to assess the amount of information on model parameters that could be encoded in the *DOT* signal.

However, with the current level of understanding, it is possible to incorporate the dynamics of the adaptation state in the models to better control cultures, to prevent oxygen depletion, to optimize glucose feeding, and to understand the influence of bolus feeding on cell behavior. The authors plan to report on that in the future.

## 6. Conclusions

A segmentation algorithm, a correlation analysis, and a mechanistic modelling approach for the analysis of the dissolved oxygen tension signal in minibioreactor systems with intermittent bolus feeding were proposed. The segmentation algorithm revealed the existence of up to four distinguishable segments in recorded *DOT* pulses, which represent the response to single substrate additions. Possible relationships between the descriptive metrics of the segments and the metabolic activities and process dynamics were investigated. The findings hypothesize a repeated metabolic switching behavior in *E. coli* after each substrate addition, where the metabolic states are linked to the identified segments of the *DOT* pulses. A newly observed *DOT* segment, not reported in the literature, is likely to be linked to a metabolic adaptation behavior. In this segment, the cell is likely to pause or attenuate the metabolism.

The quantitative analysis and the mechanistic model simulations support this hypothesis. The derived model parameter values are within acceptable ranges as determined in the literature. The mechanistic model simulations show a possibility to reproduce *DOT* segments that are found in the raw data by using parameters estimated from the quantitative analysis and by extending the model of [41] by adding a metabolic adaptation state. The duration of the proposed state can be a function of the inhibitory acetate and biomass concentration.

For our quantitative analysis, the estimation of model parameters of the overflow switching condition was possible using only a *DOT* signal and biomass samples, given that the feeding and reactor working volumes were known.

The analysis suggests that frequent metabolic switches have a negative impact on some model parameters such as the maximum oxidative uptake rate and the oxygen yields on glucose and acetate.

The results highlight the potential of considering the *DOT* signal to gain additional (unexploited) information on the *E. coli* metabolism which can be used for the estimation of cell physiological parameters. The proposed methods offer means to understand the influence of intermittent bolus feeding on cell behavior, and by that, help to address the MBR issues of oxygen supply and feeding plan optimization.

## Figures and Tables

**Table 1:** Intermittent bolus feeding plan for the eight minibioreactors. For all runs, the feeding concentration was 600 mg/mL.

Reactor Nr.	Total Feeding Volume Compared to the Reference Plan [%]	Individual Feeding Pulse Volume [µL]	Feeding Pulse Time Interval [min]	Average Dilution Ratio Per Feeding Pulse [µL/mL]
**A**	25%	5	30	0.625
**B**	50%	5.5	20	0.688
**C**	75%	6.5	12	0.813
**D**	100%	6.5	9	0.813
**E**	100%	6.5	9	0.813
**F**	125%	8.5	9	1.063
**G**	150%	6	4	0.750
**H**	175%	7.5	4	0.938

**Table 2:** Signal analysis metrics of each DOT pulse; (X) refers to the analyzed combinations.

	Metric	Segments				
		1st	2nd	3rd	4th	All
**A**	**Time length**	x	x	x	x	
**B**	**Slope**	x	x	x	x	
**C**	**Area**					x

**Table 3:** Cell physiological and additional parameters determined for each reactor.

	Cell Physiological Parameters								Additional Parameters			
Reactor Nr.	$K_{La}$		$q_{O_{2}}^{max}$		$Y_{O2/S}$		$Y_{O2/A}$		OUR		$q_{o}$	
	$\left[\%\right]$		$\left[g/\left(g\cdot h\right)\right]$		$\left[g/g\right]$		$\left[g/g\right]$		$\left[g\left(L\cdot h\right)\right]$		$\left[g/\left(g\cdot h\right)\right]$	
	−σ	+σ	−σ	+σ	−σ	+σ	−σ	+σ	−σ	+σ	−σ	+σ
**A**	180	250	0.09	0.10	0.05	0.08	0.07	0.15	0.5	0.6	0.05	0.12
**B**	200	250	0.10	0.15	0.05	0.07	0.07	0.15	0.6	0.8	0.07	0.15
**C**	220	320	0.10	0.20	0.06	0.09	0.11	0.25	1.0	2.0	0.10	0.20
**D**	200	300	0.10	0.20	0.05	0.1	0.10	0.21	1.0	2.0	0.07	0.20
**E**	200	330	0.09	0.23	0.05	0.1	0.09	0.20	1.0	2.0	0.07	0.20
**F**	190	220	0.08	0.15	0.05	0.1	0.10	0.20	1.0	1.7	0.05	0.15
**G**	180	250	0.08	0.15	0.06	0.11	0.10	0.20	1.0	3.0	0.05	0.15
**H**	160	185	0.06	0.14	0.05	0.08	0.08	0.19	1.0	2.0	0.04	0.12

**Table 4:** Volumetric mass transfer coefficient $K_{La}$, oxygen uptake rate OUR, oxygen specific uptake rate $q_{o}$, and the working volume V for *E. coli* cultivations for the MBRs systems reported in the literature.

	$K_{La}\;$	OUR	$q_{o}$	V	Notes	Literature
	$\left[1/h\right]$	$\left[g/\left(L\cdot h\right)\right]$	$\left[g/\left(g\cdot h\right)\right]$	$\left[\mathrm{mL}\right]$		
**1**	20–75	-	-	0.15	Impeller speed up to 200–800 rpm, biomass up to 6 OD (600 nm)	[35,36]
**2**	58–90	~0.5	-	1	Dry cell weight up to 0.33 g/L	[19]
**3**	90–400	-	-	6	Impeller speed 1300–1850 rpm , dry cell weight up to 1.8 g/L	[37]
**4**	400–1440	-	-	10	Impeller speed up to 2800 or 4000 rpm , dry cell weight up to 16.5 g/L	[7]
**5**	180–720	1–3.6	0.3–0.5	8–12	Impeller speed 1080–2400 rpm , dry cell weight up to 20.5 g/L	[18]
**6**	Up to 1440	-	-	8–14	Impeller speed 3000 rpm , dry cell weight up to 13 g/L	[20]
**7**	216–396	-	-	10–100	Impeller speed up to 100–7000 rpm , dry cell weight up to 10 g/L	[38,39]

**Figure A2:**
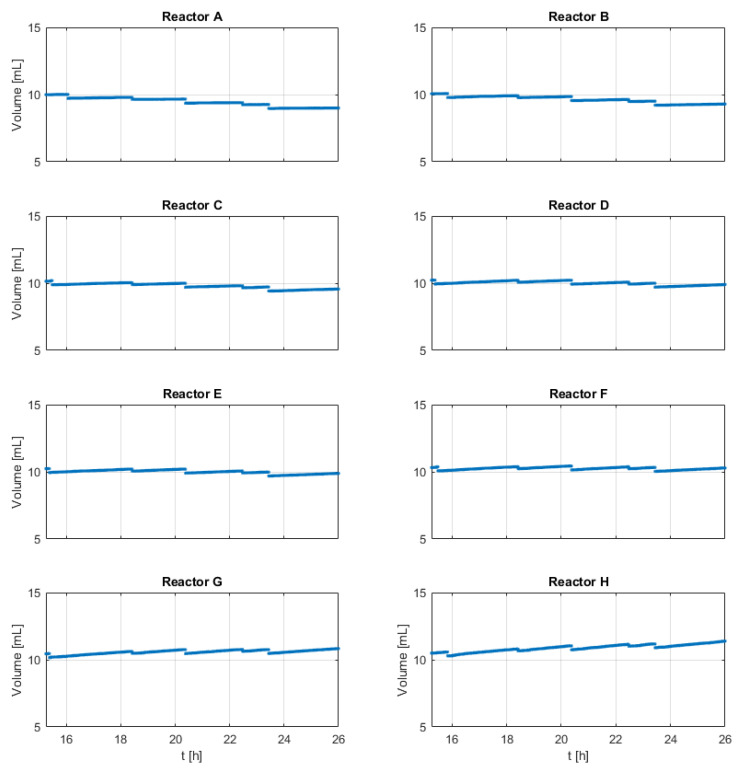
Reactor working volumes of all runs (reactors A–H).

**Figure A3:**
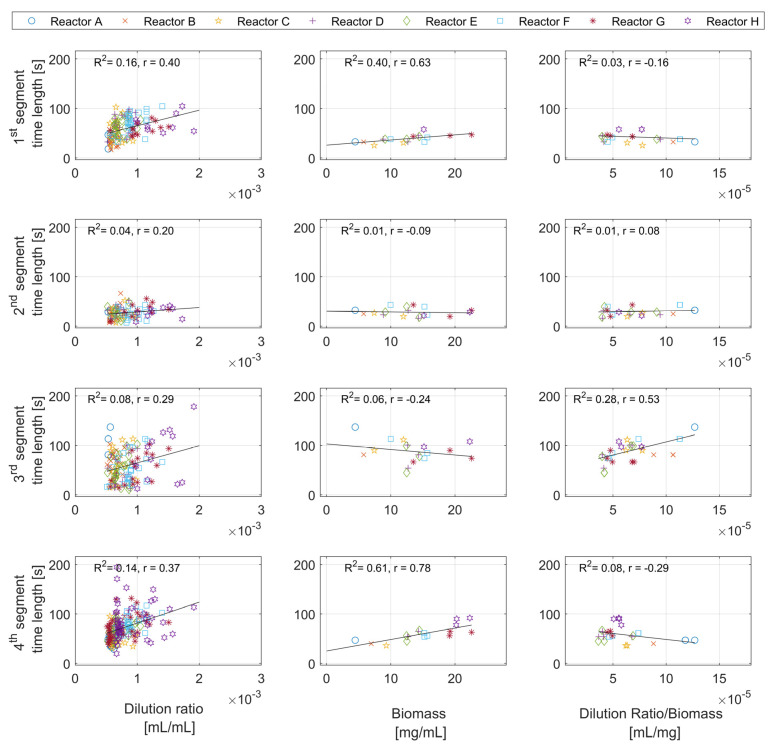
Time length of the segments against the dilution ratio, biomass concentration, and the dilution ratio per biomass concentration.

**Figure A5:**
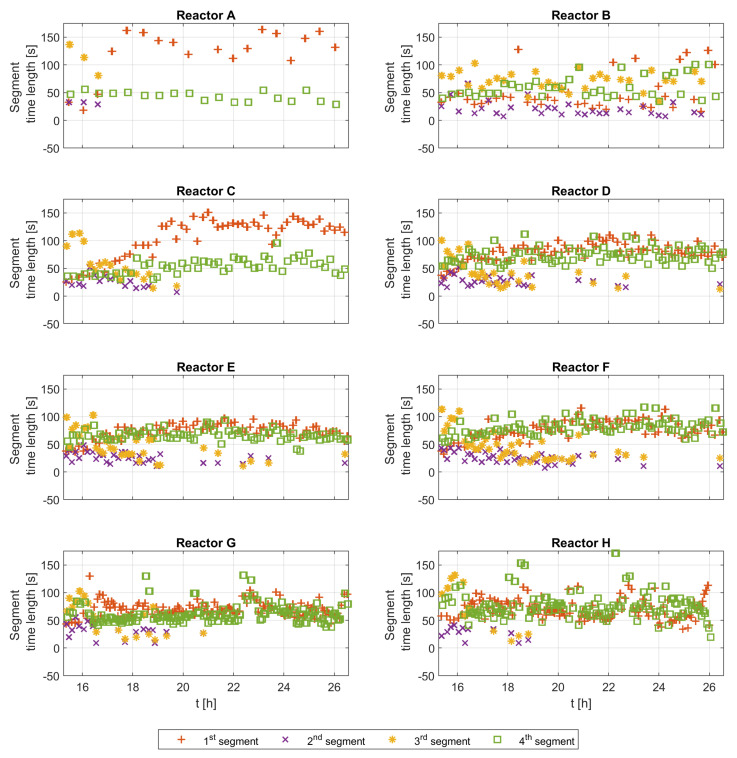
Analysis of the time length of the first, second, third, and fourth segments of all experimental runs (reactors A–H).

**Figure A6:**
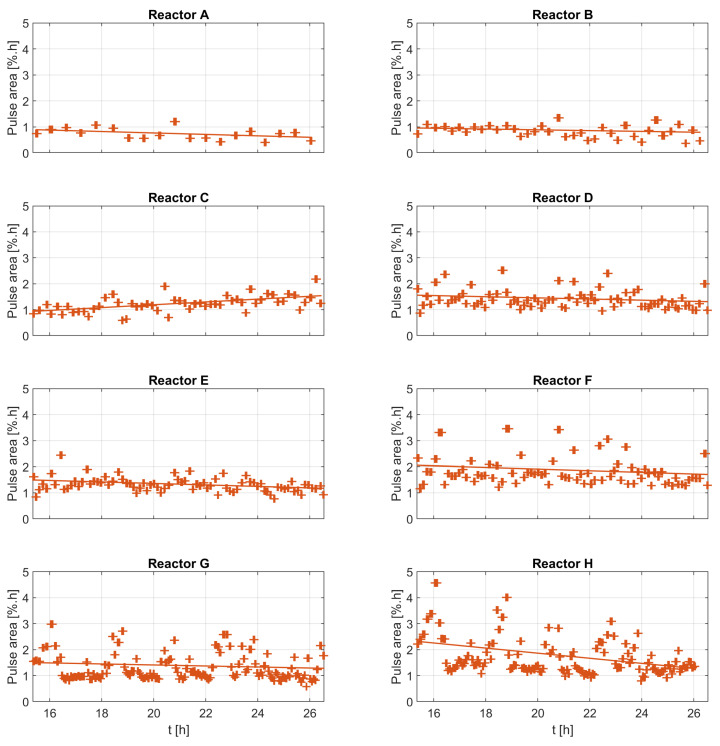
Analysis of the total area of the first, second, third, and fourth segments for all experimental runs (reactors A–H).

## Data Availability

Not applicable.

## Appendix A. Segmentation Algorithm

**Pulse detection algorithm**

The segmentation algorithm has the following steps:

(1) divide the whole signal into k ≥ 1 time windows.
(2) for each time window calculate the mean of the signal. The result is a vector containing all the means.
(3) the intersection of this vector with the *DOT* signal gives the intersection points.
(4) a loop checks whether within two consecutive intersection points the *DOT* signal has two maximums and one minimum. If so, a pulse is identified, and the time points of the two maxima define the beginning and the end times $t_{i}^{\mathrm{start}}\;\mathrm{and}\;t_{i}^{\mathrm{end}}$. The time point of the minimum is $t_{i}^{\min}$.

k represents a tuning parameter, usually set to large values for larger changes in *DOT* signals.

The result is a vector of individual *DOT* pulses P:

(A1) $$P\left(i\right)=\left[t_{i}^{start}\;;{\;t}_{i}^{min};{\;t}_{i}^{end}\right];P=\overset{n}{\underset{1}{\cup }}P\left(i\right)$$

where:

i is the index of the glucose addition (and of corresponding *DOT* pulse/response).
n is the total number of glucose pulses (and of corresponding *DOT* pulse).
$t_{i}^{start}$ is the start time of each *DOT* pulse.
$t_{i}^{min}$ is the time where the *DOT* pulse has a minimum.
$t_{i}^{end}$ is the end time of each *DOT* pulse.

**Segmentation Algorithm**

For an experimental run j, and a vector $P\left(i\right)$:

(1) split the pulse $P\left(i\right)$ into two pieces at $t_{i}^{min}$. These result in the “up-down” part (contains the first, second, and third segments) and the “down-up” part (only contains the fourth segment)
(2) for the “up-down” part, the algorithm advances along time steps and monitors the change of the slope.
(3) once a recognizable slope change $\alpha_{1}^{j}$ is observed, the algorithm determines the end of a segment and the beginning of another segment.
(4) the previous step is repeated until the three segments are found and the corresponding slopes ($\alpha_{1}^{j},\alpha_{2}^{j}$) are defined.
(5) if not all three segments are definable, assume that there is only a first “up-down” segment.

The algorithm is trained for each experimental run based on heuristics and a visual inspection using a subset of available *DOT* signals (the training dataset). The training process estimates the coefficients $\alpha_{1},\alpha_{2}$. The remaining signals are used for testing and evaluation.

## Appendix C. *E. coli* Model and Nomenclature

$$q_{s\;}=q_{s\;}^{max}\frac{C_{s}}{C_{s}+K_{s}}$$

$$q_{A\;}=q_{A}^{max}\frac{C_{A}}{C_{A}+K_{A}}$$

$$q_{O_{2}}=q_{O_{2}}^{max}\frac{DOT}{DOT+K_{O}}$$

$$q_{s\left(ox\right)\;}^{critical}=\;\;\frac{q_{O_{2}}}{Y_{O_{2}/s}}$$

$$q_{s\left(ox\right)}=\left\{\begin{array}{cc} q_{s\;} & if\;q_{s\;}\leq q_{s\left(ox\right)}^{critical}\\ q_{s\left(ox\right)\;}^{critical} & if\;q_{s\;}>q_{s\left(ox\right)}^{critical} \end{array}\right.$$

$$q_{A\left(ox\right)\;}^{critical}=\;\frac{q_{O_{2}}-Y_{O_{2}/s}\;\;\cdot \;q_{s\;\left(ox\right)}}{Y_{O_{2}/A}}$$

$$q_{A\left(ox\right)}=\left\{\begin{array}{cc} q_{A\;} & if\;q_{A\;}\leq q_{A\left(ox\right)}^{critical}\\ q_{A\left(ox\right)\;}^{critical} & if\;q_{A\;}>q_{A\left(ox\right)}^{critical} \end{array}\right.$$

$$q_{s\;\left(red\right)}=q_{s}-q_{s\left(ox\right)\;}^{critical}$$

$$\mu_{total}=Y_{x/s\left(ox\right)}.q_{s\;\left(ox\right)}\;+\;Y_{x/s\left(red\right)}\;.\;q_{s\;\left(red\right)}+\;\;Y_{x/A\left(ox\right)}\;.\;q_{A\;\left(ox\right)}$$

$$\frac{d}{dt}\left[\begin{array}{c} C_{x}\\ C_{s}\\ C_{A}\\ DOT \end{array}\right]=\left[\begin{array}{ccccc} Y_{x/s\left(ox\right)} & Y_{x/s\left(ox\right)} & Y_{x/s\left(red\right)} & Y_{x/A\left(ox\right)} & Y_{x/A\left(ox\right)}\\ -1 & -1 & -1 & 0 & 0\\ 0 & 0 & Y_{e/s} & -1 & -1\\ -Y_{\frac{O_{2}}{s}} & -Y_{\frac{O_{2}}{s}} & 0 & -Y_{\frac{O_{2}}{A}} & -Y_{\frac{O_{2}}{A}} \end{array}\right]\cdot \;A\cdot \left[\begin{array}{l} q_{s}\\ q_{s\left(ox\right)}^{critical}\\ q_{s\;\left(red\right)}\\ q_{A}\;\\ q_{A\left(ox\right)}^{critical} \end{array}\right]C_{x}\;-\frac{F_{s}}{\;V}\;\left[\begin{array}{c} C_{x}\\ C_{s}\\ \begin{array}{c} C_{e}\\ 0 \end{array} \end{array}\right]+K_{La}\left[\begin{array}{c} 0\\ 0\\ \begin{array}{c} 0\\ DOT* \end{array} \end{array}\right]-K_{La}\left[\begin{array}{c} 0\\ 0\\ \begin{array}{c} 0\\ DOT \end{array} \end{array}\right]$$

**Table A1:** Activation matrix values for metabolic states defined in Section 4.2.

Metabolic State/Active Submodel	Activation Matrix A
(I) Overflow metabolism	$A=diag\;\left[0\;1\;1\;0\;0\right]$
(II) Adaptation state	$A=diag\;\left[0\;0\;0\;0\;0\right]$
(III) Acetate oxidation	$A=diag\;\left[0\;1\;0\;0\;1\right]$
(IV) Static state	$A=diag\;\left[0\;0\;0\;0\;0\right]$

Sampling and feeding volumes are considered as sets of algebraic equations solved outside the ODE system. The timepoints of these pulses are considered as explicit time events, and for a greater explanation see [31,41]. The value of the $q_{m}$, the specific maintenance coefficient term, is assumed to be zero purposes of simplification. This value is not relevant to the scope of this contribution.

**Table A2:** Model parameters, description, and values.

PAR.	Unit	Simulation Values	Description
$\mu_{max}$	$h^{-1}$	-	Maximum growth rate
$q_{S}^{max}$	$g\left(s\right).g{\left(x\right)}^{-1}.h^{-1}$	2.7	Maximum specific glucose uptake rate of the Monod function
$q_{O_{2}}^{max}$	$g\left(O\right).g{\left(x\right)}^{-1}.h^{-1}$	0.15	Maximum specific oxygen uptake rate of the Monod function
$q_{A}^{max\;}$	$g\left(A\right).g{\left(x\right)}^{-1}.h^{-1}$	0.8	Maximum specific acetate uptake rate of the Monod function
$q_{s\left(ox\right)\;}^{critical}$	$g\left(O\right).g{\left(x\right)}^{-1}.h^{-1}$	-	Maximum specific glucose uptake rate defined by the maximum oxidative capacity
$q_{A\left(ox\right)\;}^{critical}$	$g\left(A\right).g{\left(x\right)}^{-1}.h^{-1}$	-	Maximum specific acetate uptake rate by the maximum oxidative capacity
$q_{s\left(ox\right)}$	$g\left(s\right).g{\left(x\right)}^{-1}.h^{-1}$	-	Actual specific glucose uptake rate
$q_{A\left(ox\right)}$	$g\left(A\right).g{\left(x\right)}^{-1}.h^{-1}$	-	Actual specific acetate uptake rate
$Y_{X/S\left(red\right)}$	$g\left(x\right).g{\left(s\right)}^{-1}$	0.4	Biomass yield for reductive growth on glucose
$Y_{X/S\left(ox\right)}$	$g\left(x\right).g{\left(s\right)}^{-1}$	0.5	Biomass yield for oxidative growth on glucose
$Y_{X/A\left(ox\right)}$	$g\left(x\right).g{\left(A\right)}^{-1}$	0.5	Biomass yield for oxidative growth on acetate
$Y_{A/S}$	$g\left(A\right).g{\left(s\right)}^{-1}$	0.4	Acetate yield from glucose fermentation
$Y_{O2/A}$	$g\left(O_{2}\right).g{\left(A\right)}^{-1}$	0.5	Oxygen (stoichiometric) yield on acetate
$Y_{O2/S}$	$g\left(O_{2}\right).g{\left(s\right)}^{-1}$	0.1	Oxygen (stoichiometric) yield on glucose
$K_{la}$	$h^{-1}$	225	Oxygen mass transfer coefficient from the gas phase to the liquid phase
$K_{A}$	$g\left(A\right).L^{-1}$	0.001	Time affinity constant of the acetate
$K_{S}$	$g\left(S\right).L^{-1}$	0.001	Time affinity constant of the glucose
$K_{O}$	$g\left(O_{2}\right).L^{-1}$	0.001	Time affinity constant of the oxygen
***H***	$\%/g\left(O_{2}\right).L^{-1}$	14000	Henry Law derived constant
$C_{X}$	$g\left(X\right).L^{-1}$	-	Biomass concentration
$C_{S}$	$g\left(S\right).L^{-1}$	-	Glucose concentration
$C_{A}$	$g\left(A\right).L^{-1}$	-	Acetate concentration
DOT	%	-	Dissolved oxygen tension
$DOT_{m}$	%	-	Measured Dissolved oxygen tension
***DOT****	%	-	Dissolved oxygen tension at saturation
τ	h	0.01	Dissolved oxygen probe response time
***V***	L	-	Working volume
***OUR***	$g\left(O2\right).L^{-1}.h^{-1}$	-	Oxygen uptake rate
***OTR***	$g\left(O2\right).L^{-1}.h^{-1}$	-	Oxygen transfer rate

## Appendix D. Data-Driven Analysis

The Bartlett statistical tests for all runs show high values ($x^{2}\approx$ [650, 1800] with p-value=0), and the ANOVA test also shows high values for all runs (F≈ [337, 3100] with p-value=0), which indicate a difference between in the time segments of each run.
